# Campylobacter jejuni-associated perimyocarditis: two case reports and review of the literature

**DOI:** 10.1186/s12879-016-1635-7

**Published:** 2016-06-14

**Authors:** Fredrik Hessulf, Johan Ljungberg, Per-Anders Johansson, Mats Lindgren, Johan Engdahl

**Affiliations:** Department of Anaesthesiology and Intensive Care Medicine, Hallands Hospital, Halmstad, Sweden; Department of Internal Medicine, Hallands Hospital, Halmstad, Sweden; Department of Molecular and Clinical Medicine/Cardiology, Sahlgrenska Academy, University of Gothenburg, Gothenburg, Sweden

**Keywords:** Campylobacter jejuni, Perimyocarditis, High-sensitive Troponin T, ST-elevation

## Abstract

**Background:**

*Campylobacter spp.* are among the most common bacterial causes of gastroenteritis world-wide and mostly follow a benign course. We report two cases of *Campylobacter jejuni*-associated perimyocarditis, the first two simultaneous cases published to date and the third and fourth cases over all in Sweden, and a review of the literature.

**Case presentation:**

A previously healthy 24-yo male (A) presented at the Emergency Department(ED) with recent onset of chest pain and a 3-day history of abdominal pain, fever and diarrhoea. The symptoms began within a few hours of returning from a tourist visit to a central European capital. Vital signs were stable, the *Electrocardiogram*(ECG) showed generalized ST-elevation, laboratory testing showed increased levels of C-reactive protein(CRP) and high-sensitive Troponin T(hsTnT). Transthoracic echocardiogram (TTE) was normal, stool cultures were positive for *C Jejuni* and blood cultures were negative. Two days after patient A was admitted to the ED his travel companion (B), also a previously healthy male (23-yo), presented at the same ED with almost identical symptoms: chest pain precipitated by a few days of abdominal pain, fever and diarrhoea. Patient B declared that he and patient A had ingested chicken prior to returning from their tourist trip. Laboratory tests showed elevated CRP and hsTnT but the ECG and TTE were normal. In both cases, the diagnosis of *C jejuni*-associated perimyocarditis was set based on the typical presentation and positive stool cultures with identical strains. Both patients were given antibiotics, rapidly improved and were fully recovered at 6-week follow up.

**Conclusion:**

Perimyocarditis is a rare complication of *C jejuni* infections but should not be overlooked considering the risk of heart failure. With treatment, the prognosis of full recovery is good but several questions remain to be answered regarding the pathophysiology and the male preponderance of the condition.

## Background

*Campylobacter spp.* are among the most common bacterial causes of gastroenteritis world-wide and usually the result of handling and consumption of poultry meat [1]. Typical symptoms include diarrhoea, fever and abdominal cramps. Infections are sometimes asymptomatic, often self-limiting and rarely require antibiotics. Bacteraemia is seen in less than 1 % of cases, acute and post-infectious complications are rare but include arthritis, meningitis, endocarditis, sepsis and Guillain-Barré syndrome [2, 3]. Myocarditis and perimyocarditis are known but rare complications of *C jejuni* infections. Here we present an unusual case where two previously healthy males presented at the ED with diarrhoea and chest pain and where the diagnosis of *C jejuni*-associated perimyocarditis was set. We also present the results of a review of the literature and discuss disease characteristics i.e., the preponderance of young healthy males and pathophysiological mechanisms.

## Case presentation

### Case 1

**Fig. 1:**
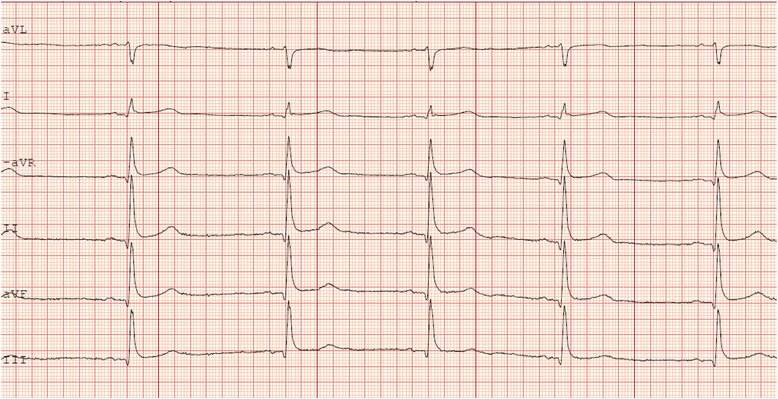
Limb leads from 12-lead ECG from patient A showing typical generalized ST-segment elevation

A previously healthy 24-year-old male (A) presented at the emergency department (ED) with chest pain and diarrhoea. The chest pain began 2 h prior to hospital admission and was described as a constant light chest pressure without correlation to breathing or body position. 3 days earlier, the patient and a friend had had chicken in a restaurant before boarding a flight to Sweden. One hour after arrival in Sweden the patient experienced sudden abdominal pain, chills and diarrhoea. During the following three days the patient had 6 diarrhoeas per day, mucous but no visible blood. The patient was diagnosed with *C jejuni* gastroenteritis after stool culture at a primary care facility, no antibiotics were given. Upon admission to the ED the abdominal pain had subsided, the patient was afebrile (37 °C or 98.6 °F) but still experiencing diarrhoea. Upon examination the patient had normal circulatory and respiratory parameters. Heart auscultation: regular rhythm (RR), no murmurs or extra sounds. Lung and abdominal examinations were normal. Laboratory examinations showed C-reactive protein (CRP) at 89.1 (normal range <10 mg/L), leukocyte count (LC) at 11.3 (normal range 4-12 × 10^9^/L) and high-sensitive Troponin T (hsTnT) at 108 (normal range <14 ng/L) ECG showed regular sinus rhythm, 64/min and general 1 mm ST-elevation (Fig. 1). The patient was treated with Brufen 200 mg (tid), Omeprazol 20 mg (qd) and Loperamid 2 mg and admitted to a cardiac care unit (CCU) for cardiac monitoring.

During the following 4 days the hsTnT reached a maximum value of 504 and then dropped to 46. Stool culture confirmed the diagnosis of *C jejuni*. Blood cultures were negative. Transthoracic echocardiogram (TTE) showed normal right and left ventricle function, ejection fraction (EF) 60–65 %, normal valvular structure and function, no hypokinesia or pericardial effusion. After on day the ECG-changes had resolved. The patient was started on ciprofloxacin but developed urticarial rashes and severe itching and the treatment was discontinued. The chest pain subsided after 2 days and the patient left the hospital after 4 days. The patient was given a 10 day prescription of Azithromycin 500 mg (qd) upon hospital release. The diagnosis was determined to be *C jejuni*-associated perimyocarditis (CPM). At follow-up visit 4 weeks after discharge the patient was without complaints, physical examination was normal and both ECG and TTE were normal.

### Case 2

Two days after A was admitted to the ED with chest pain, his travelling companion (B) presented on the same ED with a 48-h history of mild constant chest pain exacerbated by breathing but not body position. B had, in the same fashion as his companion A, developed abdominal pain, diarrhoea and chills upon return to Sweden. After initially experiencing multiple diarrhoeas daily, upon admission to the ED the diarrhoeas had started to subside and the abdominal pain resolved. The patient was afebrile (37.4 ° C or 99.3 °F), circulatory and respiratory parameters as well as heart, lung and abdominal examination were normal. Laboratory tests showed CRP at 46.5, LPK at 9.1 and hsTnT at 128. The ECG was normal. The patient was admitted to a CCU for hsTnT-serial testing and cardiac monitoring.

During the hospital stay the hsTnT reached a maximum of 128 before dropping to 52. Stool cultures confirmed the diagnosis of *C jejuni* sensitive to Azithromycin. Blood cultures were negative. TTE showed normal right and left ventricle function, normal valvular structure and function and no pericardial effusion. The chest pain resolved within 48 h and the patient was released from the hospital with a 10-day prescription of Azithromycin 500 mg qd. The patient was given the diagnosis of *C jejuni*-associated perimyocarditis (CPM). At follow-up visit 4–6 weeks after discharge the patient was doing well, physical examination, stress ECG and TTE were all normal.

## Discussion

*C jejuni* infections are common but rarely lead to severe symptoms outside of the gastrointestinal tract [2]. To date only a few case reports [4, 5] have described *C jejuni*-associated myocarditis/perimyocarditis (CPM) despite the fact that *C jejuni* infections are one of the most common causes of gastroenteritis word wide. In this case report we present an unusual case of two previously healthy young males who, after eating chicken at a restaurant, develop typical gastroenteritis symptoms (abdominal pain, diarrhoea, chills) within 24 h and within 48–72 h of the onset of gastroenteritis symptoms also develop chest pain. Based on elevated hsTnT, typical ECG-findings (case A), positive stool cultures, a lack of alternative plausible explanations and a temporal association, the diagnosis of *C jejuni*-associated perimyocarditis was set. Subsequent analysis of both stool cultures demonstrated the presence of the same *C jejuni* strains with identical genotypes. At follow up 4–6 weeks after discharge the patient in case one was asymptomatic, the patient in case 2 complained of unspecific fatigue that had resided 3 weeks later. Both patients had normal TTE and stress ECG examinations.

This case report highlights several points of interest regarding pathogenesis and epidemiology of campylobacter-associated perimyocarditis. Here follows a review of the literature and further discussion of the case report.

A search on MEDLINE/PubMed using any combination of the MESH-terms “Campylobacter”, “Campylobacter jejuni” “myocarditis”, “perimyocarditis” and “pericarditis” yielded 34 relevant articles 1979–2015. After cross checking the reference lists two additional articles were found. Including the characteristics of the two case reports presented above(Case A and B), a total of 44 cases; 20 cases of myocarditis, 19 cases of perimyocarditis and five cases of pericarditis associated with *C jejuni* infection have been described in the literature. We review the baseline characteristics of the patients based on the published case reports (Table 1), and summarize the most recent case reports in more detail (Table 2), review the clinical presentation of *C jejuni* myocarditis, differential diagnosis and diagnostic issues, treatment, pathophysiology and certain disease characteristics.

**Table 1:** Summary of baseline characteristics of all published case reports including patient A and patient B. *n* = 44 [4–6, 10–12, 14, 18, 22–49]^a,b^

Characteristics		
Age (years)	Mean	29,4
	Max	60
	Min	15
Myocarditis *n* (%)	All	20(100)
	Female	0(0)
	Male	20(100)
Perimyocarditis *n* (%)	All	19(100)
	Female	1(5)
	Male	18(95)
Pericarditis *n* (%)	All	4(100)
	Female	3(75)
	Male	1(25)

^a^Campylobacter not identified to the species level (ref [26] and [28])

^b^Mean age (43 years old) of the patient series which included one case of Campylobacter (ref [28])

**Table 2:** Summary of the most recent case reports including case 1 (patient A)^a^ and 2 (patient B)^b^. *n* = 14

Sex/Age	Cardiac marker	ECG	Echo	MRI	Antibiotics	Outcome
M/24^a^	TnT 504 ng/L	ST-elevation	Normal, LVEF 60–65 %	No	Azithromycin	Full recovery
M/23^b^	TnT 128 ng/L	Normal	Normal incl LVEF	No	Azithromycin	Full recovery
M/60 [6]	N/A	N/A	Pericard effusion	No	Ceftriaxone/Meropenem	Diarroea
M/43 [7]	TnT 1.75 ng/ml	ST-elevation	Abn wall motion LVEF 68 %	Subepi/myocardial enhancement	Azithromycin	N/A
M/33 [8]	TnI 18.6mcg/L	ST-elevation	LVEF 56 %	Increased signal subepi, dilated LV/RV	Roxithromycin	N/A
M/17 [9]	TnI 16.8 ng/mL	ST-elevation	Normal	Subepicardial enhancement	Azithromycin	Full recovery
M/21 [5]	TnI 15.6 ng/ml	Normal	Decreased LVEF	Subepi/myocardial enhancement	Azithromycin	Almost full rec
M/24 [5]	TnI 8.9 mcg/L	ST-elevation	LVEF 40 %	Subepicardial enhancement	No	Full recovery
M/42 [5]	TnI 11.6mcg/L	ST-elevation	LVEF 40 %	Subepicardial enhancement	Ciprofloxacin	Full recovery
M/21 [10]	TnI 39.8mcg/L	ST-elevation	Normal inkc LVEF	No	Ciprofloxacin	Full recovery
M/21 [11]	TnI 2.5mcg/L	ST-elevation	Normal incl LVEF	No	Yes(unknown kind)	Full recovery
M/24 [12]	CK normal	Arrythmia	Decreased LVEF/peric effusion	No	Roxithromycin	N/A
M/19 [13]	TnT 0.52 ng/mL	Strain	N/A	Yes	N/A	N/A
M/16 [14]	TnT 1.7 ng/mL	ST-elevation	LVEF 45 %	No	Clarythromycin	Full recovery
M/17 [14]	TnT 0.9 ng/mL	ST-elevation	Normal incl LVEF	Myocardial enhancement	Clarythromycin	Full recovery

The mean age was 29 years, 93 % of cases affected men. Few patients had multiple comorbidities, a majority of patients were previously healthy, but there were also a substantial amount of cases that affected immunocompromised patients.

The clinical presentation is variable and there are no pathognomonic symptoms or signs. Typically, the patient presents with a history of a few days of gastroenteritis (fever, abdominal pain, diarrhoea) and recent onset of chest pain/discomfort/tightness. Vital signs are often in the normal range. The ECG often shows signs of tachycardia and generalized ST-elevation/depression/T-wave inversion, easily mistaken for an acute coronary syndrome resulting in normal coronary angiography examinations. Additional differential diagnosis includes valvular pathology and pulmonary embolism. Laboratory investigations generally show elevated levels of cardiac enzymes (Troponin T/I or Creatinine Kinases MB Isoenzyme (CKMB) as well as inflammation markers (CRP). Additional diagnostic measures have traditionally included TTE and, more recently, cardiac Magnetic Resonance Imaging (MRI). The TTE can sometimes reveal decreased left ventricular ejection fraction (LVEF), wall motion abnormalities and pericardial effusion. Cardiac MRI is a non-invasive method that has gained popularity lately. Subepicardial and myocardial enhancement can be seen primarily in the left ventricle. Based on the combination of symptoms (abdominal and chest symptoms) and signs (ECG-changes and laboratory investigations) the diagnosis of myocarditis is suspected and the patient admitted to a cardiac care unit (CCU) for continuous cardiac monitoring. The cardiac monitoring is often uneventful but at least on case of severe arrhythmia (ventricular tachycardia) has been described [6]. Historically, endomyocardial biopsy (EMB) has been performed and is considered the gold standard for diagnosing myocarditis. However, there is a risk of complications related to the procedure (although probably less common than previously thought [7]. According to the AHA/ACCF/ESC joint statement [8] and a recent review [9] EBM should be performed in patients with severe symptoms (heart failure, dilated cardiomyopathy, hemodynamic compromise) where the risks are outweighed by the potential benefits. Pena et al. [10] reported a rare case of *C jejuni*-associated myocarditis with pathological evidence of inflammation (but no evidence of *C jejuni* on polymerase chain reaction (PCR) study of the myocardium).

Treatment can be divided into supportive and causal. Supportive treatment consists of preventing dehydration and electrolyte disturbances and managing eventual heart failure (Angiotensin receptor blocker/B adrenergic blocker) or arrhythmias. Causal treatment consists of antibiotic treatment; primarily Macrolides (Azithromycin/Roxithromycin/Clarythromycin) and Flouroquinolones (Ciprofloxacin) have been used. To the best of our knowledge there is no consensus on dosage and treatment duration; when reviewing recent cases the most common choice of antibiotic was the Macrolide Azithromycin, 500 mg qd for 3–10 days [11] Full recovery and return to normal physical status was noted in a vast majority of published cases. Complications include persistent heart failure [12] and ventricular arrhythmia [6]. Few cases with severe and fatal outcome have been described in the literature, and all but one had pre-existing conditions [10].

This case report highlights 3 points of interest.

1. We are, to our knowledge, the first to type a *C jejuni* strain associated with myocarditis. Using Pulse-field gel electrophoresis (PFGE) and the restriction enzyme SMAL the two campylobacter isolates showed identical patterns (Fig. 2) and the same pattern of antibiotic resistance. No match of a similar strain could be found in the Swedish database of *C jejuni* strains, and the only similar strain we could find was from a Swiss chicken isolate from 2008 and this isolate was not associated with any disease. The two cases in this report are also the first ever described where the same *C jejuni* strain caused myocarditis in two related cases. All previous case reports have reported single cases of myocarditis. This raises the question whether different campylobacter strains have different affinity for the myocardium. Our two cases represent the third and fourth case of myocarditis ever described in Sweden, which highlights the rare nature of the condition.

**Fig. 2:**
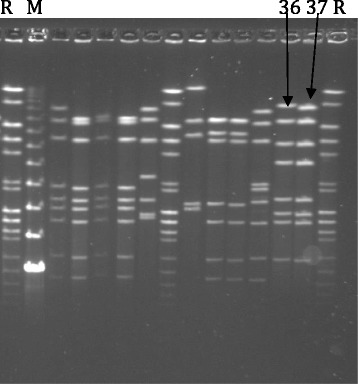
Pulsed-field gel electrophoresis according to the standardized Campynet protocol and using *Sma*I restriction enzyme. R: normalisation standard; M: molecular size marker; 36 and 37 show the banding patterns of the *C. jejuni* isolates from patient A and B, respectively
2. Our case report also highlight the need for further investigation of the mechanisms of campylobacter-associated myocarditis. Earlier studies have speculated that there might be at least two types of CPM: one bacteria/toxin mediated that causes acute symptoms within 2–4 days of the gastroenteritis and another, immunological type, that causes delayed symptoms 2 weeks after the onset of the infection [13]. Neither type has been particularly well characterized, there is to our knowledge only one case of *C jejuni*-myocarditis with pathological confirmation [10]. Alzand et al. speculate that several mechanisms, apart from direct bacterial invasion, such as bacterial toxins, circulating immune complexes or cytotoxic T-cells might be involved [14]. In 2007 Becker et at studied a Danish cohort of 6204 cases of *C jejuni*-gastroenteritis and found no increase in myocarditis incidence in the *C jejuni* cohort and no cases of pericarditis as compared to age matched controls. Because of the patient selection, retrospective study design and the very low incidence of myocarditis (1/6204) and pericarditis (0/6204) in the *C jejuni*-cohort the authors limited their conclusion to state that they did not observe an increased incidence of myocarditis or pericarditis. The authors offered an intriguing but speculative hypothesis for the aetiology of campylobacter-associated myocarditis: the possibility of a viral co-infection that might be the actual cause of the perimyocarditis/myocarditis.
  Because of the relatively acute onset of chest pain in relation to the debut of the infection, one might speculate that the most probable cause of myocarditis in this case report is a bacteria/toxin effect. The fact that blood cultures were negative does not rule out transient bacteraemia or a toxin effect. Less than 1 out of 100 Campylobacter gastroenteritis infections have positive blood cultures [15] which have led some to believe that we under-diagnose *Campylobacterimia* [16]. A recent study by Harvala et al. showed an increase in the number of Campylobacteremia cases in Sweden in 2014 as a result of improvements in blood culture medium [17] This fact could explain why, in the presently described as well as in all previously described cases of myocarditis/perimyocarditis/pericarditis apart from one case of pericarditis [18], blood cultures have been negative. This patient was also immunocompromised due to an X-linked Agammaglobulinemia, and one can speculate that this could affect the disease mechanism.
3. This case report and literature review also highlights the fact that (young) men seem to be affected by campylobacter-associated myocarditis/perimyocarditis to a larger extent than women. The incidence of *C jejuni* gastroenteritis is slightly higher for men than women but almost all reported CPM have affected men [2]. This observation has been made several times before, but no explanation has been offered. If one combines all reported cases of myocarditis and perimyocarditis associated with *C jejuni* infection described in the literature to date, only 7 % affect women (4/43). A comparison with the incidence of other bacteria-associated types of myocarditis is difficult because of lack of reliable studies. It is a well-known fact that most types of cardiovascular disease have a higher incidence and prevalence among men than women (at least up to the age of 75) [19], and recent reports [20] have shown and confirmed a higher incidence of myocarditis in men as compared to women. The exact mechanisms for this is not known but some authors speculate that factors such as sex hormones (both testosterone as well as the female sex hormones), the immune system and genomics as well as differences in clinical manifestation and treatment might play a role [21].

## Conclusion

Myocarditis and perimyocarditis are rare complications of *C jejuni* infections that predominantly affect young previously healthy men and in the majority of cases follow a benign course. More studies are needed to elucidate the characteristics and pathogenesis of this rare condition.

## Abbreviations

CKMB, creatinine kinase MB isoenzyme; CPM/CM/CP, campylobacter jejuni-associated perimyocarditis/myocarditis/pericarditis; CRP, C-reactive protein; ECG, electrocardiogram; ED, emergency department; EMB, endo-myocardial biopsy; hsTnT, high sensitive troponin; LC, leukocyte count; LVEF, left ventricle ejection fraction; MRI, magnetic resonance imaging; PCR, polymerase chain reaction; PFGE, pulse-field gel electrophoresis; T C jejuni, campylobacter jejuni; TTE, transthoracic echocardiogram
